# Supplementary material for: Acquisition of molecular rolling lubrication by self-curling of graphite nanosheet at cryogenic temperature
Source: Nat Commun. 2024 Jul 9;15:5747. doi: 10.1038/s41467-024-49994-4 (PMC11233547; doi:10.1038/s41467-024-49994-4)
Supplement: Supplementary file 1 — Supplementary Information [file 41467_2024_49994_MOESM1_ESM.docx]

Supplementary information

**Acquisition of molecular rolling lubrication by self-curling of graphite nanosheet at cryogenic temperature**

Panpan Li^1,2#^, Wenhao He^1#^, Pengfei Ju^3^, Li Ji^1,2*^, Xiaohong Liu^1^, Fan Wu4, Zhibin Lu^1,2*^, Hongxuan Li^1,2*^, Lei Chen^1,2^, Jingzhou Liu^3^, Huidi Zhou^1,2^ & Jianmin Chen^1,2*^

^1^State Key Laboratory of Solid Lubrication, Lanzhou Institute of Chemical Physics, Chinese Academy of Sciences, Lanzhou 730000, China

^2^Center of Materials Science and Optoelectronics Engineering, University of Chinese Academy of Sciences, Beijing 100049, China

^3^Shanghai Aerospace Equipment Manufacture, Shanghai 200245, China

^4^Changchun Institute of Applied Chemistry, Chinese Academy of Sciences, Changchun, 130022, China

^#^The authors contributed equally.

*E-mails: jili@licp.cas.cn (Lead contact); zblu@licp.cas.cn; lihx@licp.cas.cn; chenjm@licp.cas.cn

**This PDF file includes:**

[Supplementary Note 1: Microstructure of the frictional interface 2](#_Toc167743829)

[Supplementary Note 2: Friction and wear behavior of graphite at different temperatures 3](#_Toc167743830)

[Supplementary Note 3: The influence of graphite intrinsic structure (defects, orientation) 4](#_Toc167743831)

[Supplementary Note 4: The influence of counterpart activity 6](#_Toc167743832)

[Supplementary Note 5: Influences of defects on the formation of graphite nanorollers 10](#_Toc167743833)

[Supplementary Note 6: Influences of temperature on the morphology of GNSs 11](#_Toc167743834)

[Supplementary Note 7: Structural evolution of the nanoroller during dynamic friction process 12](#_Toc167743835)

[Supplementary Note 8: Influence of low temperature on the microstructure of GNSs 13](#_Toc167743836)

[Supplementary Note 9: The influence of number of layers on self-curling phenomenon 14](#_Toc167743837)

[Supplementary Note 10: Models and set-up for DFT calculations 16](#_Toc167743838)

[Supplementary Note 11: Schematic diagram of ultralow temperature frictional equipment 16](#_Toc167743839)

[Supplementary Note 12: Model and set-up for MD simulation 16](#_Toc167743840)

[References 17](#_Toc167743841)

**Supplementary Note 1: Microstructure of the frictional interface**

The original graphite surface and the wear scars after the friction test at different temperature (50 K and 300 K) were characterized by Raman spectroscopy (Supplementary Figure 1). The intensity of the *D* peak of the original graphite is slightly low, and it increases significantly after friction. Especially after friction test at 50 K, the intensity of the *D* peak is higher than that of the *G* peak, and new peaks emerge at 218 cm^-1^ and 283 cm^-1^, corresponding to the radial breathing mode of *sp*^2^ carbon^1,2^. Besides, the new peak at 804 cm^-1^ also corresponds to the formation of the graphite nanorollers after firiction at ultralow temperature in vacuum.

**Supplementary Fig. 1| Raman spectra of original graphite and the wear scars after the friction test at different temperatures in vacuum.**

**Supplementary Note 2: Friction and wear behavior of graphite at different temperatures**

The morphologies of wear scars and wear tracks of graphite after sliding against GCr15 steel ball at different temperatures (300 K and 50 K) in vacuum are shown in Supplementary Figure 2. It can be seen that transfer film is accumulated on the friction surface of the counterpart steel ball and large wear scar is formed after the friction test at 300 K, which corresponds to the serious wear (the wear track width is about 1098 µm) thereat. While after the friction test at 50 K, a uniformly transfer film is formed on the friction surface of the counterpart steel ball and the wear scar width is greatly reduced (about 351 µm), corresponding to significantly reduced wear.


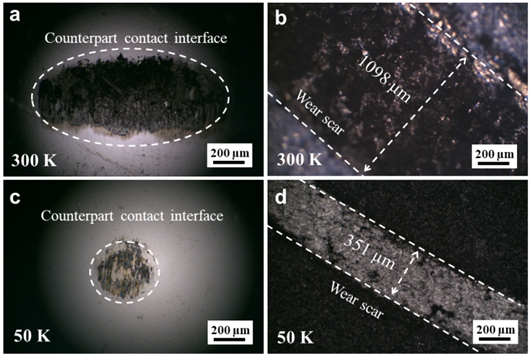


**Supplementary Fig. 2| Optical images of wear scars and wear tracks after friction test at different temperatures in vacuum.** (**a**) Wear scar at 300 K. (b) Wear track at 300 K. (**c**) Wear scar at 50 K. (**d**) Wear track at 50 K.

**Supplementary Note 3: The influence of graphite intrinsic structure (defects, orientation)**

The intrinsic structural characteristics of graphite (and pyrolytic graphite) are discussed further to explore the influence. Considering the influence of graphite defect on the rolling process, the pyrolytic graphite with fewer defects and better basal plane orientation is introduced for comparison. Supplementary Figure 3 shows the Raman spectra of graphite and pyrolytic graphite, respectively. They both possess typical *D* peak, *G* peak and *2D* peak, and the *D* peak intensity of pyrolytic graphite is lower, which corresponds to its fewer original defects. Besides, the original graphite is disordered, being a typical polycrystalline. As shown in Supplementary Figure 4, graphite shows an evident diffraction peak at around 25°, which corresponds to the (002) crystal basal plane of graphite. The diffraction peaks at around 42°, 44°, 54°and 77° are attributed to the (100), (101), (004) and (110) crystalline planes of graphite. The three evident diffraction peaks at around 26°, 54° and 86° are attributed to the (002), (004) and (006) crystalline planes of pyrolytic graphite respectively. These XRD data illustrates that the basal plane of pyrolytic graphite is of better orientation than the one of graphite. In addition, pyrolytic graphite has better lamellar orientation than graphite, while the surficial orientation of graphite is disordered (Supplementary Figure 5). Viewing these differences in the microstructure of the two types of graphite, both graphite and pyrolytic graphite can form graphite nanorollers on the friction interface at 50 K (Supplementary Figure 6), and more numerous and widespread nanorollers are formed on the friction interface of pyrolytic graphite, which indicates that GNSs with perfect original structure (fewer defects and better orientation of basal plane) are more favorable for the formation of nanorollers. Because the pyrolytic graphite possesses fewer defects and better orientation as well as larger nanosheets, its layers are easier to be peeled off and the as-formed nanorollers are not as intact as those of graphite with smaller nanosheets.

**Supplementary Fig. 3| Raman spectra of graphite and pyrolytic graphite. (a)** Graphite. **(b)** Pyrolytic graphite.

**Supplementary Fig. 4| XRD pattern of graphite and pyrolytic graphite. (a)** Graphite. **(b)** Pyrolytic graphite.


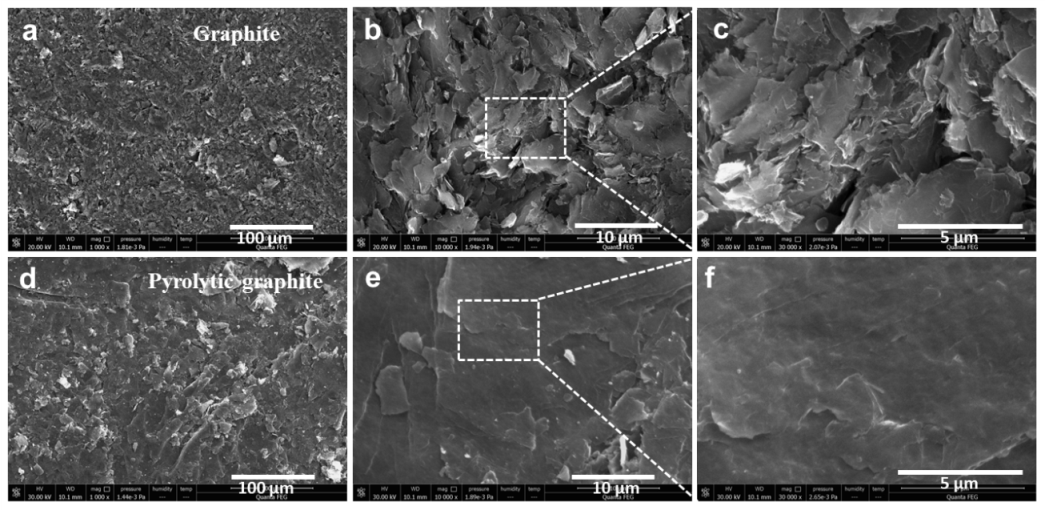


**Supplementary Fig. 5| Original surface morphology of graphite and pyrolytic graphite. (a-c)** Graphite. **(d-f)** Pyrolytic graphite.


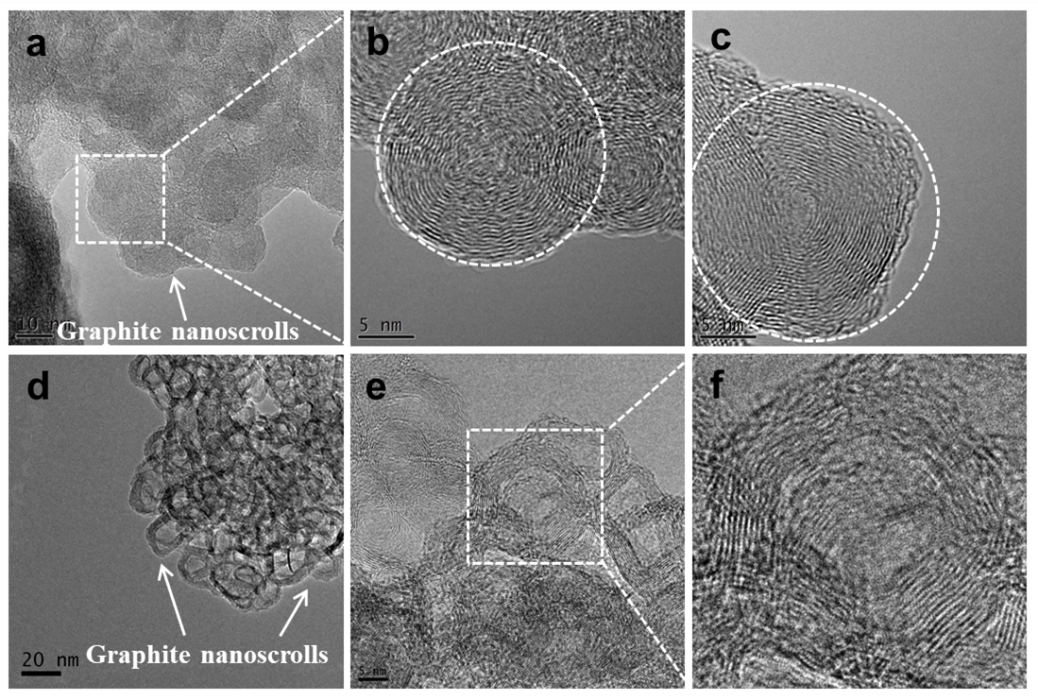


**Supplementary Fig. 6| The microstructure of friction interface of graphite and pyrolytic graphite sliding against counterpart GCr15 steel ball at 50 K**. (**a-c**) TEM and HRTEM images of graphite of friction interface after friction. (**d-f**) TEM and HRTEM images of pyrolytic graphite of friction interface after friction.

**Supplementary Note 4: The influence of counterpart activity**

Supplementary Figure 7 is the cross-sectional microstructure of counterpart GCr15 steel ball and the wear track after friction test at 300 K, and it shows that CNSs with an inclination of about 45° to the friction direction, indicating the crystal lattice orientation of the graphite basic planes is not parallel to the friction direction, but the edges are exposed on the friction surface, and its inside local lattice is deformed and distorted. The transfer film formed on the steel ball can be divided into two layers, one is the contact slip interface layer of amorphous carbon with a thickness of about 2 ~ 3 nm, another is the layer of amorphous carbon wrapped with iron carbide crystallites and graphite fragments. It is manifested that strong chemical interaction occurs between the graphite and the counterpart steel ball as well during the friction process at 300 K in vacuum, causing severe damage to the original graphite flakes in association with the generation of broken graphite fragments with exposed edge dangling bonds and defects. Therefore, the graphite does not follow the sliding mechanism, and the high energy fragments with exposed edge dangling bonds and defects exhibit strong adhesion to the friction steel surface, leading to high friction. While at 50 K (Supplementary Figure 8), the lubricating film on the wear track spreads evenly, and an ordered layer is formed on the contact friction interface. It is shown that the lattice orientation of the graphite basic plane is parallel to the contact surface while graphite nanorollers emerge at the subsurface. In addition, only accumulated and transferred graphite crystallite fragments are found inside the cross-sectional microstructure of the counterpart steel ball, but no iron carbide crystallites are observed. The electron diffraction (ED) of the friction steel surface shows that the transfer film consists of graphite without iron carbide phase (Supplementary Figure 8d), which indicates that no chemical interaction occurs between the graphite and counterpart steel ball during friction at of 50 K in vacuum, namely the cryogenic temperature inhibits the chemical activity of the counterpart steel ball. Namely, the interaction between graphite and the counterpart steel ball is greatly inhibited at cryogenic temperature in vacuum, and hence no iron carbide is produced (different from the case at normal temperature; Supplementary Figure 7).

**
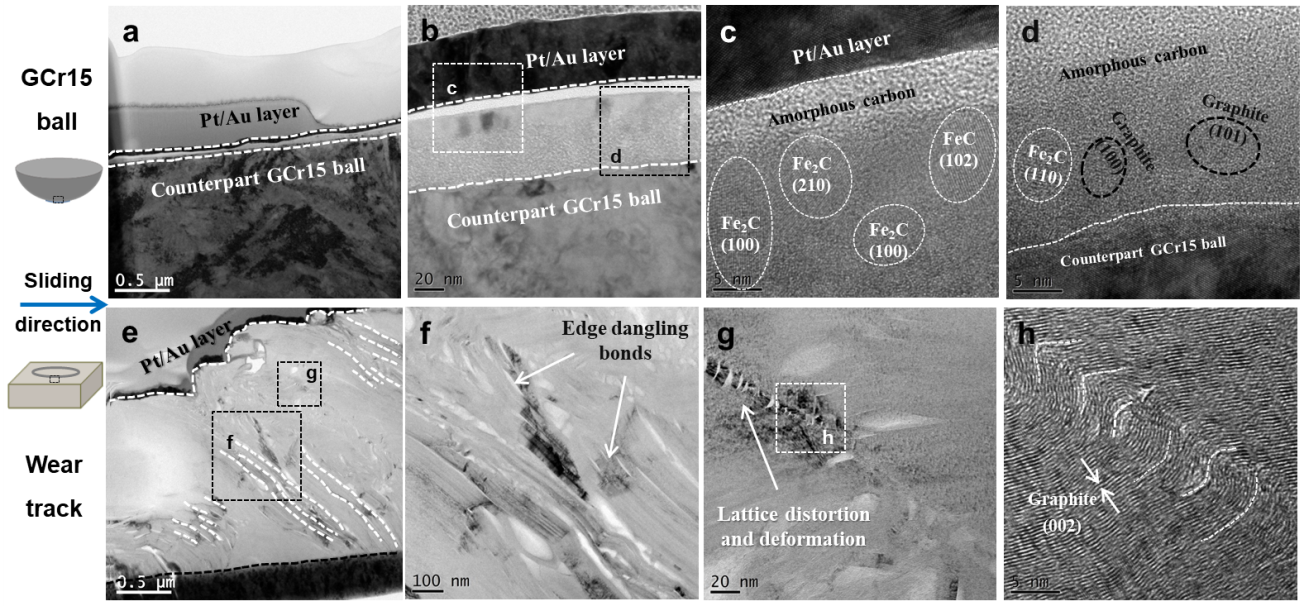
**

**Supplementary Fig. 7| Cross-sectional microstructure of counterpart GCr15 steel ball and the wear track after friction test at 300 K.** (**a-d**) Cross-sectional microstructure and HRTEM images of wear scar of counterpart GCr15 steel ball. (**e-h**) Cross-sectional microstructure and HRTEM images of wear track of graphite.


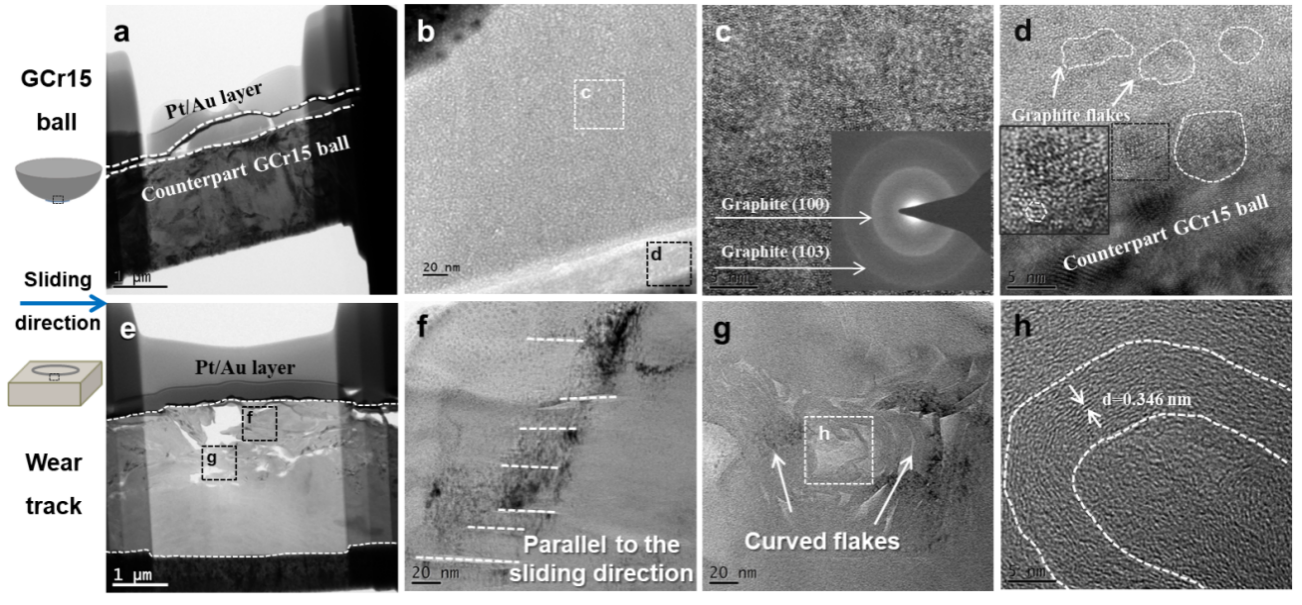


**Supplementary Fig. 8| Cross-sectional microstructure of counterpart GCr15 steel ball and the wear track after friction test at 50 K.** (**a-d**) Cross-sectional microstructure and HRTEM images of wear scar of counterpart GCr15 steel ball. (**e-h**) Cross-sectional microstructure and HRTEM images of wear track of graphite.

When friction test is conducted at 300 K, a short-term low-friction state is observed. Corresponding TEM images of the friction interface show that an ordered layer-layer sliding structure is formed after a short time of friction (Supplementary Figure 9a and b), and Supplementary Figure 10c is the schematic. The as-formed ordered layer-layer structure allows the molecular sliding mechanism to exert, and the friction coefficient is relatively low (about 0.09 ~ 0.10). However, after a long period of friction, the GNSs are damaged to form amorphous carbon in association with few broken fragments. The orientation of broken graphite flakes is disordered; and the crystal lattice is deformed, due to the exposed edge of dangling bonds and defects interact strongly (Supplementary Figure 9d and e), and Supplementary Figure 9f shows the corresponding schematic. There are not any nanorollers formed during the whole friction process of graphite slid against counterpart GCr15 steel ball at 300 K in vacuum. As a result, the friction coefficient is high and fluctuates.


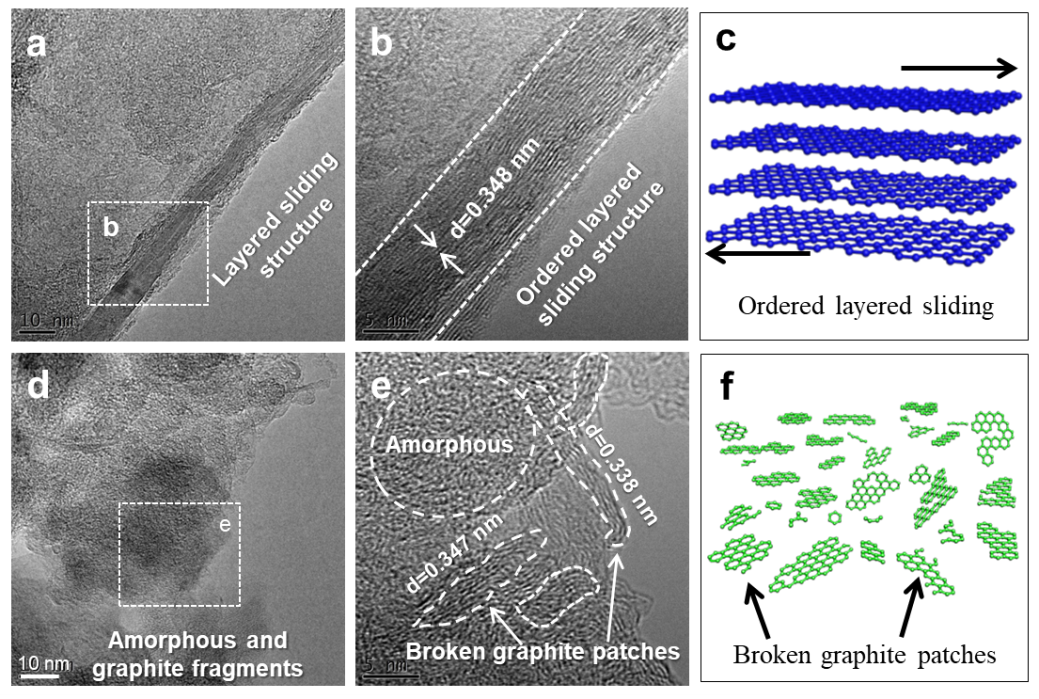


**Supplementary Fig. 9| The microstructural evolution of frictional interface at different stages of graphite slid against GCr15 counterpart steel ball at 300 K**. **(a-c**) The TEM and HRTEM images of the friction interface as well as the schematic of the microstructure after a short time friction (about 4 minutes). (**d-f**) The TEM and HRTEM images of the friction interface as well as the schematic of the microstructure after long time friction (about 45 minutes).

Supplementary Figure 10 shows the morphology of the wear scars and the wear tracks after the friction test at different temperatures of 300 K and 50 K in vacuum when graphite slid against counterpart Al_2_O_3_ ball. It can be seen that the uniformly spread transfer film is formed on the counterpart ball. Both the contact area of wear scars are corresponded to the width of the wear track. The width of wear track is about 505.2 µm at 300 K and slightly larger than 310.2 µm at 50 K, which indicates the wear at 50 K is slightly lower than at 300 K. When the Al_2_O_3_ counterpart ball with weak chemical activity is used as the counterpart to assemble the friction pair with graphite, the microstructure of friction interface demonstrates that the nanorollers are formed only at the cryogenic temperature, which is favorable for the graphite to exhibit excellent tribological behaviour at cryogenic temperature in vacuum. Thus, the activity of the counterpart ball has insignificant influence on the formation of nanorollers, while the applying cryogenic temperature is essential.

The morphology of the friction interface at the stable friction stage shows that an ordered layer-layer sliding structure is formed after friction at 300 K in vacuum (Supplementary Figure 11a-c); and the formed ordered layer-layer sliding structure exhibits layered sliding mechanism thereat. Different from this, graphite nanorollers are formed at 50 K (Supplementary Figure 11d-f). It demonstrates that the counterpart balls with different chemical activity do not influence the formation of the graphite nanorollers and the graphite nanorollers can form by friction at cryogenic temperature.


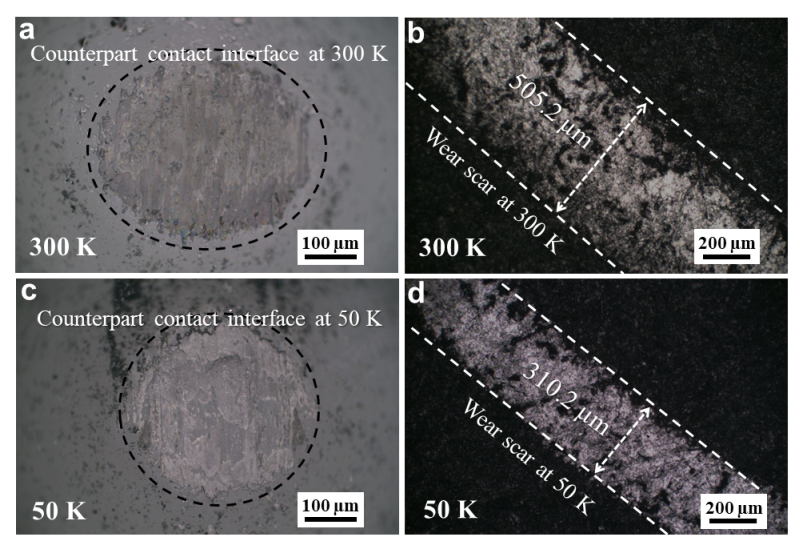


**Supplementary Fig. 10| Optical images of wear scars and wear tracks after friction test of graphite slid against counterpart Al_2_O_3_ ball at different temperatures.** (**a**) Wear scar at 300 K. (**b**) Wear track at 300 K. (**c**) Wear scar at 50 K. (**d**) Wear track at 50 K.


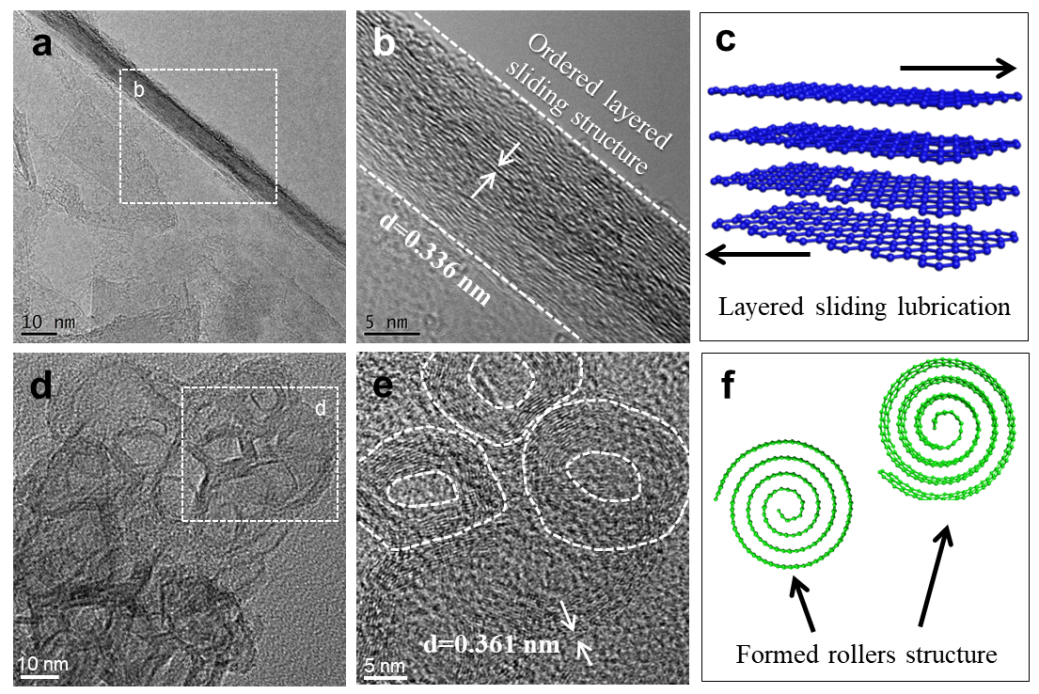


**Supplementary Fig. 11| The microstructure of friction interface of graphite slid against counterpart Al_2_O_3_ ball at different temperatures in vacuum.** (**a-c**) TEM and HRTEM images of the friction interface as well as the schematic diagram of the microstructure of the friction interface at 300 K. (**d-f**) TEM and HRTEM images of the friction interface as well as the schematic of the microstructure of the friction interface at 50 K.

**Supplementary Note 5: Influences of defects on the formation of graphite nanorollers**

The influence of defects on the the formation of graphite nanorollers was investigated by introducing 0.5 at% vacancies into the CNSs exposed to temperature gradient from 300 K to 70 K. As shown in Supplementary Figure 12, the graphite nanorollers formed finally and the curling behavior of the GNS remains nearly unchanged, which indicates that the influence of the vacancies on the initial graphite nanoroller formation is negligible. Furthermore, Supplementary Figure 13 shows the graphite nanorollers formation process during dynamic friction at 100 K with 0.5 at% vacancy defects introduced into the nanosheets after the initial curl edge structure is formed. It is noted that C-C bonds are formed between the defects and edge dangling bonds with high energy at 100 K. Nevertheless, the formed C-C bonds weaken and inhibit the rolling as well as the formation of graphite nanorollers, thus the formation of graphite nanorollers are restrained. Therefore, defects are adverse for the formation of intact graphite nanorollers during the friction process.


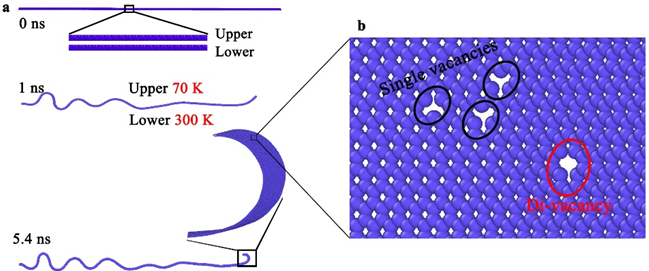


**Supplementary Fig. 12| MD simulation of the formation of curled edge for GNSs with 0.5 at% defects under temperature gradient from 300 K to 70 K.** (**a**) Formation of the initial graphite nanoroller under different temperature fields (300 K and 70 K). (**b**) Original defects on the CNSs.


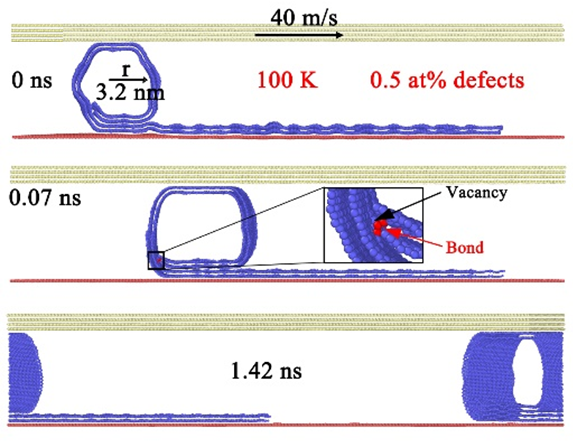


**Supplementary Fig. 13| The nanorollers formation for graphite with 0.5 at% defect during friction process in different temperature after the formation of the initial graphite nanoroller at 100 K.** All atoms marked with different colors are C atom.

**Supplementary Note 6: Influences of temperature on the morphology of GNSs**

To explore the atomic structural changes of graphite nanosheets at cryogenic temperature, the liquid nitrogen treatment and *in-situ* cryogenic TEM observation is conducted (Supplementary Figure 14). Since graphite participates in the rolling process in the form of nanosheets (Supplementary Figure 9), GNSs is selected; and the *in-situ* nanodeformation of the GNSs before and after cryogenic temperature treatment is investigated by TEM. After being immersed in liquid nitrogen for 1 h, the GNSs show obvious edge curling along the long-edge (Supplementary Figure 14a and 14b). The morphologies of GNSs before *in-situ* cryogenic temperature treatment show that the two pieces of GNSs in the sight are stacked together at 300 K (Supplementary Figure 14c), and the edge regions of the nanosheets are overlapped in association with the presence of wrinkles ascribed to the inherent lattice fluctuation thereat. As the sample bottom is cooled by liquid nitrogen for equilibrium, the two pieces of GNSs are no longer stacked together but separated with a certain distance, and their edges are curled and folded (Supplementary Figure 14d). Corresponding high resolution transmission electron microscopy (HRTEM) image of the GNS on the right side shows that the edge is the (002) basal lattice plane of graphite with vertical configuration rather than the (001) lattice plane with original horizontal configuration (Supplementary Figure 14e and 14f), which indeed demonstrates the GNSs are curled at cryogenic temperature.

**Supplementary Fig. 14| The *in-situ* TEM microstructural variation of GNSs upon varying temperature.** (**a**-**b**) TEM images of GNSs before and after immersing in liquid nitrogen (77 K). (**c**-**f**) *In-situ* TEM images of GNSs at 300 K and 77 K.

**Supplementary Note 7: Structural evolution of the nanoroller during dynamic friction process**

The structural evolution of the nanorollers formation during friction process is shown in Supplementary Figure 15, which can be divided into three processes. The sample is graphite block with multi-layers. In the process 1, at the initial friction stage, the friction force between the steel ball and graphite surface is higher than the van der Waals force between graphite interlayers, and thus graphite is delaminated and scratched to form graphite nanosheets with several layers. In the process 2, the delaminated graphite nanosheets cover on the surface of graphite blocks. The upper surface of the graphite nanosheets is exposed to a vacuum environment of 300 K, while the lower surface contacts the graphite block at 50 K. The temperature difference between the upper and lower surfaces of the graphite nanosheet drive the self-curling of the graphite nanosheet edge. In the process 3, under the action of shear force, the self-curling graphite nanosheet is further curled to form intact and parallel nanoroller layer-by-layer.

**Supplementary Fig. 15| Schematic of the nanoroller formation during experimental friction process.**

**Supplementary Note 8: Influence of low temperature on the microstructure of GNSs**

Supplementary Figure 16 shows the *in-situ* cooling XRD spectra of the GNSs treated by liquid nitrogen during the temperature-declining process. It can be seen that XRD peaks of GNSs shifts to high diffraction angle with the temperature decreases, demonstrating the in-plane lattice constant and the interlayer spacing of the GNSs at cryogenic temperature are smaller than that of normal temperature. In other words, the atomic distance of the GNSs tends to decrease after immersion in liquid nitrogen with the temperature decreases; and, thus, any unbalanced factors such as structural defects and temperature alternation during the cooling process would cause changes of atomic distance and uneven atomic shrinkage to produce stress of the GNSs, thereby leading to their curling deformation. The curling deformation of GNSs under temperature gradient would induce the change of the optical and electronic properties; and the temperature-induced nanodeformation behavior of GNSs could be of particular significance for its application in sensing and other fields^3-5^.

**Supplementary Fig. 16| The *in-situ* XRD spectra of CNSs during varying temperature.**

**Supplementary Note 9: The influence of number of layers on self-curling phenomenon**

Supplementary Figure 17 shows that the graphite nanosheet is curled for one cycle under the force $F$ caused by the temperature gradient field, which is equivalent to the top layer shrinking by $2\pi\left( n-1 \right)d$, and the work done by the force is

$W_{F}=F*2\pi\left( n-1 \right)d$ (1)

where $n$ is the number of layers, $d$ is the interlayer spacing and $F=\tau*l*b$ holds. $\tau$ is the stress caused by the temperature gradient field. $\text{l}$ and $\text{b}$ is respectively the length and width of the graphite nanosheet. Therefore, it can be obtained that

$W_{F}=\tau*l*b*2\pi(n-1)d$ (2)

Meanwhile the deformation energy of graphite nanosheet rolled for one cycle is

$W_{\mathrm{def}}=\frac{1}{2}D*{(\frac{1}{R})}^{2}\text{*}2\pi\text{R*b}=D\frac{\pi\text{b}}{R}$ (3)

where, $R$ is bending radius and $D$ is the bending stiffness, which relies significantly on the number of layers^6^. Here, by fitting with the power function, the $D$ of the graphite nanosheet within 10 layers are obtained (Supplementary Figure 17b).

To bend the graphite nanosheet, the work done by the force $F$ caused by the temperature gradient field should be greater than the deformation energy, that is $W_{F}\geq W_{\mathrm{def}}$, namely,

$\text{τ}\geq\frac{D}{2Rld}*\frac{1}{n-1}$ (4)

Therefore, the stresses of graphite nanosheet with 2 and 3 layers are 3.1 MPa and 6.1 MPa, respectively (Supplementary Figure 17c), when a graphite nanosheet with length of 2.46 μm and R is about 5 nm. With the number of layers increases, the internal stress required becomes greater, making it less likely to curl only by the temperature gradient. While internal stress to curl graphite nanosheet with 8 layers reaches 37.7 MPa, which is difficult to curl. And the low thermal conductivity makes it difficult for graphite nanosheet with 8 layers to form a uniform temperature gradient, which further hinders the process of temperature gradient driving it to curl (Supplementary Figure 18).

**
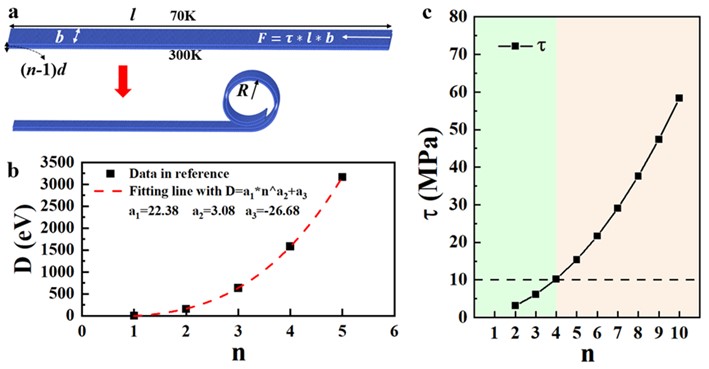
**

**Supplementary Fig. 17| The critical stress to curl the graphite nanosheet.** (**a**) Schematic diagram of the graphite nanosheet from plane to curling at edges. The length, width and thickness of the graphite nanosheet are $l$, $b$ and $(n-1)d$ respectively, where $n$ is the number of layers and $d$ is the interlayer spacing. (**b**) Bending stiffness curve of graphite nanosheet with number of layers. Short dotted line is the fitting of the experiment data with $D=a_{1}*n^{a_{2}}+a_{3}$. (**c**) The critical stress curve to curl the graphite nanosheet with the number of graphite nanosheet layers. The values of $l$ and $R$ is from the MD simulations, which is consistent with the experimental result in magnitude.


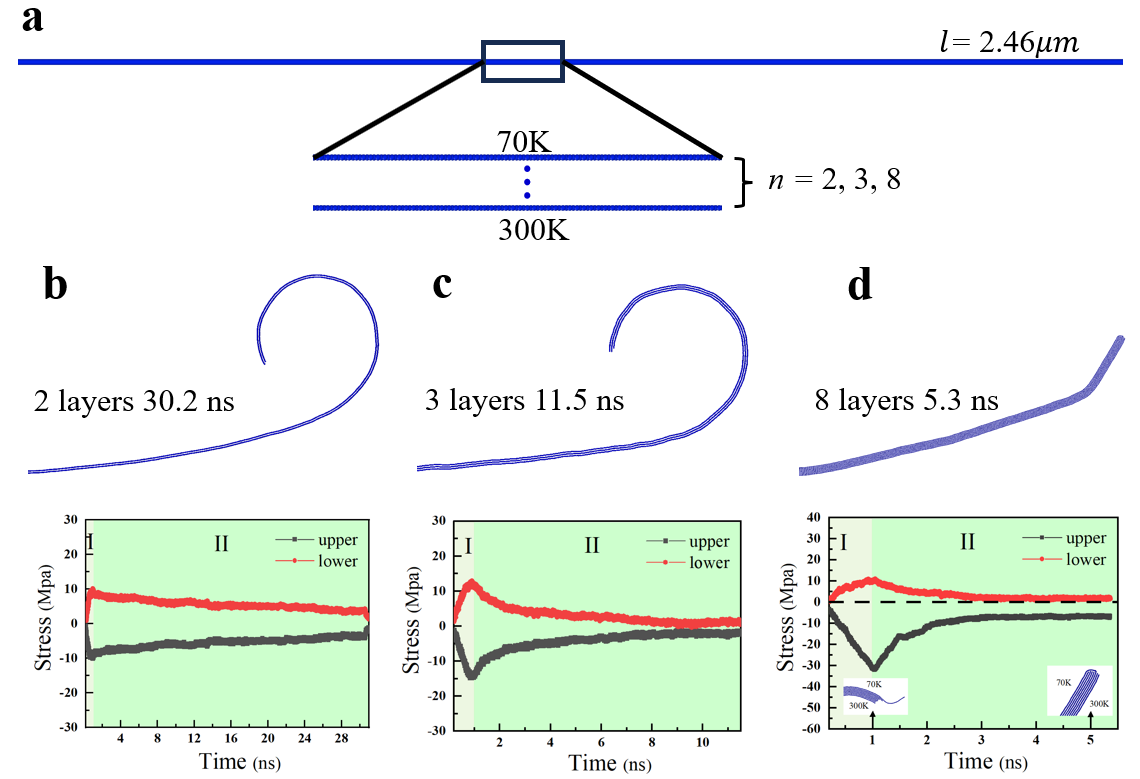


**Supplementary Fig. 18| The curling at edges of graphite nanosheets with different layers.** (**a**) Models used in molecular dynamics simulations. The final state and corresponding stress evolution of the curling at edges of 2-layer graphite nanosheet (**b**), 3-layer graphite nanosheet (**c**) and 8-layer graphite nanosheet (**d**). An unexpected result is that the stress on the top layer of the 8-layer graphite nanosheet reaches 30 MPa, and the stress on the bottom layer is still about 10 MPa. By careful analysis of the structure, it can be seen that there was abnormal elongation at the top layer, which did not completely contract until about 5 ns, as shown in the insert in **d**. This resulted in a residual stress of about 10 MPa in the upper layer even when the lower layer stress was almost completely released. Therefore, it is a reasonable assumption that the effective stress is still about 10 MPa in 8-layer graphite nanosheet.

**Supplementary Note 10: Models and set-up for DFT calculations**


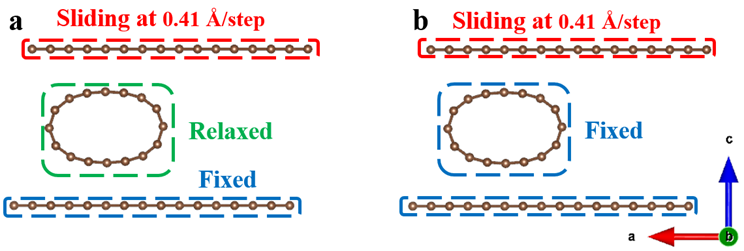


**Supplementary Fig. 19| The systems used to simulate the rolling (a) and sliding (b).**

**Supplementary Note 11: Schematic diagram of ultralow temperature frictional equipment**


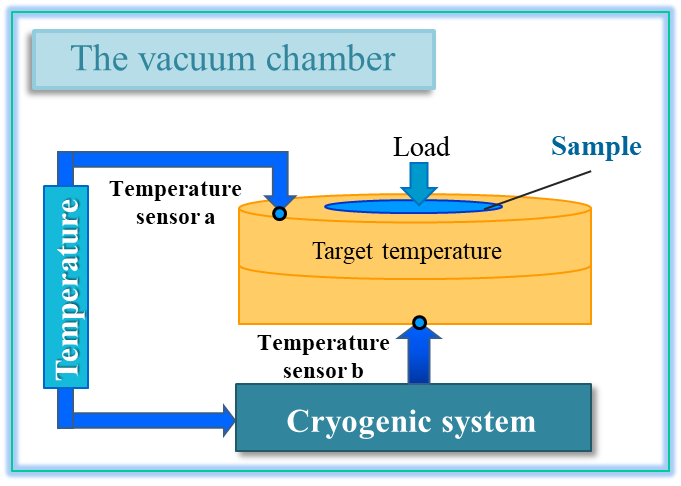


**Supplementary Fig. 20| Schematic of the friction test machine of low-temperature in vacuum.**

**Supplementary Note 12: Model and set-up for MD simulation**


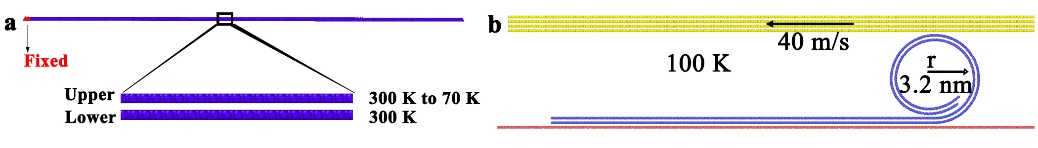


**Supplementary Fig. 21| The diagrams of the simulation systems for the structural evolution of the graphite nanoroller under temperature gradient field**. (**a**) Under friction conditions. (**b**) During friction process. All atoms marked with different colors are C atom. The red atoms are fixed in **a**. The yellow, blue and red atoms are respectively graphite, graphite nanoscroll and graphite nanosheet in **b**.

**Supplementary References**

1. Saito, R., Hofmann, M., Dresselhaus, G., Jorio, A, & Dresselhaus, M. S. Raman spectroscopy of graphene and carbon nanotubes. *Adv. Phys.***60**, 413-550 (2011).

2. Jorio, A., Souza, & Filho, A. G. Raman studies of carbon nanostructures. *Annu. Rev. Mater. Res.* **46**, 357-382 (2016).

3. Zhao, B*.* et al. High-order superlattices by rolling up van der Waals heterostructures. *Nature* **591**, 385-390 (2021).

4. Deng, T*.* et al. Three-dimensional graphene field-effect transistors as high-performance photodetectors. *Nano Lett* **19**, 1494-1503 (2019).

5. Wu, Y., Zhao, X., Shang, Y., Chang, S., Dai, L., & Cao, A. Application-driven carbon nanotube functional materials. *ACS Nano* **15**, 7946-7974 (2021).

6. Zhang, D. B. Akatyeva, E. & Dumitrică, T. Bending ultrathin graphene at the margins of continuum mechanics. *Phys. Rev. Lett.* **106**, 255503 (2011).
